# Selective importance of the rat anterior thalamic nuclei for configural learning involving distal spatial cues

**DOI:** 10.1111/ejn.12409

**Published:** 2013-11-11

**Authors:** Julie R Dumont, Eman Amin, John P Aggleton

**Affiliations:** 1 School of Psychology, Cardiff University70 Park Place, Cardiff, CF10 3AT, Wales, UK

**Keywords:** anterior thalamic nuclei, biconditional learning, configural learning, hippocampus, spatial discrimination

## Abstract

To test potential parallels between hippocampal and anterior thalamic function, rats with anterior thalamic lesions were trained on a series of biconditional learning tasks. The anterior thalamic lesions did not disrupt learning two biconditional associations in operant chambers where a specific auditory stimulus (tone or click) had a differential outcome depending on whether it was paired with a particular visual context (spot or checkered wall-paper) or a particular thermal context (warm or cool). Likewise, rats with anterior thalamic lesions successfully learnt a biconditional task when they were reinforced for digging in one of two distinct cups (containing either beads or shredded paper), depending on the particular appearance of the local context on which the cup was placed (one of two textured floors). In contrast, the same rats were severely impaired at learning the biconditional rule to select a specific cup when in a particular location within the test room. Place learning was then tested with a series of go/no-go discriminations. Rats with anterior thalamic nuclei lesions could learn to discriminate between two locations when they were approached from a constant direction. They could not, however, use this acquired location information to solve a subsequent spatial biconditional task where those same places dictated the correct choice of digging cup. Anterior thalamic lesions produced a selective, but severe, biconditional learning deficit when the task incorporated distal spatial cues. This deficit mirrors that seen in rats with hippocampal lesions, so extending potential interdependencies between the two sites.

## Introduction

The rodent anterior thalamic nuclei are thought to be critical for spatial learning by acting in concert with the hippocampus (Sutherland & Rodriguez, 1989; Warburton *et al*., 2000, 2001; Henry *et al*., 2004). The present study examined this relationship by comparing the importance of the anterior thalamic nuclei for biconditional learning when that learning involves either proximal contextual cues or more distal spatial cues. The rationale arises from the finding that hippocampal lesions can have contrasting effects on biconditional problems involving these same two cue types, with only the latter condition impaired (Coutureau *et al*., 2002; Sziklas & Petrides, 2002; Dumont *et al*., 2007; Albasser *et al*., 2013). The question is, therefore, whether anterior thalamic lesions produce a similar dissociation when tested using tasks that produce this hippocampal dissociation.

Biconditional discriminations take the general form that when stimulus A is associated with X there is one outcome, but when stimulus A is associated with Y there is a different outcome. The opposite outcomes are linked to stimulus B creating the counterbalanced arrangement AX+, AY−, BX−, BY+. One rationale for studying biconditional problems is that they help isolate specific stimulus and specific response combinations involved in learning. This property is highlighted by studies showing that anterior thalamic lesions impair only some biconditional discriminations (Sziklas & Petrides, 1999; Gibb *et al*., 2006). For example, anterior thalamic lesions impair learning to select object A (but not B) when in the North of a maze and to select object B (but not A) when in the South (Sziklas & Petrides, 1999). Examples of spared biconditional learning include making a correct motor response (turn left, turn right) depending on which object (A or B) is present (Sziklas & Petrides, 1999; see also Chudasama *et al*., 2001; Ridley *et al*., 2002; Sziklas & Petrides, 2004, 2007).

The present study had two goals. The first was to test whether anterior thalamic lesions produce the same profile of performance as seen after hippocampal damage. The second was to identify the nature of any observed deficits. Two cohorts of rats were examined. Cohort 1 first received two biconditional discriminations that involved acquiring different associations between one of two auditory stimuli with one of two test chambers distinguished by either their thermal (warm versus cool) or visual (spot versus checkered) surfaces (Ward-Robinson & Honey, 2000). These same biconditional tasks seem unaffected by hippocampal lesions (Coutureau *et al*., 2002). The next two biconditional discriminations involved (i) learning to dig in a specific cup when in a particular room location (distal spatial cues) and (ii) learning to dig in a specific cup when in one of two distinctive test boxes (local cues). Hippocampal lesions only disrupt the distal cue task (Albasser *et al*., 2013). Cohort 2 was then trained on two go/no-go place discriminations. These digging tasks tested whether rats with anterior thalamic lesions could distinguish between two locations, and then whether they could master a biconditional problem that relied on discriminating the same locations.

## Materials and methods

### Subjects

Two separate cohorts of male Lister Hooded rats were used (cohort 1, *n *=* *25 and cohort 2, *n *=* *26). The rats weighed 270–320 g at the beginning of the experiment (cohort 1, Harlan, Bicester, UK, and cohort 2, Charles River, Kent, UK) and were housed in pairs under a 12-h light–dark cycle. The animals were given free access to water but were maintained at 85% of their free-feeding weight for the duration of the experiments. The rats received either bilateral lesions of the anterior thalamic nuclei (ATN; cohort 1, ATNx1, *n *=* *15; cohort 2, ATNx2, *n *=* *14) or sham surgeries (Sham1, *n *=* *10; Sham2, *n *=* *12). All animals were habituated to handling before the start of the first experiment. All experiments were performed in accordance with the UK Animals (Scientific Procedures) Act (1986) and associated guidelines. These procedures were also approved by the appropriate ethics committee at Cardiff University.

### Surgery

Surgery was performed under pentobarbitone sodium anaesthesia (60 mg/kg i.p.; Sigma-Aldrich Company Ltd, Dorset, UK). Once anaesthetised, the animal was placed in the head-holder of the stereotaxic apparatus (Kopf Instruments, CA, USA) with the incisor bar adjusted to +5.0 relative to the horizontal plane. Following an incision, the scalp was retracted to expose the skull. A craniotomy was made and the dura cut, exposing the cortex above the target location. Lesions to the anterior thalamic nuclei were made by injecting 0.12 m *N*-methyl-d-aspartic acid (NMDA; Sigma Chemicals UK) dissolved in sterile phosphate buffer (pH 7.4) over two separate sites within one hemisphere with the use of a 1-μL Hamilton syringe (Hamilton, Switzerland) attached to a stereotaxic frame. The lateral (0.22 μL) and medial (0.24 μL) sites were infused with NMDA over a period of 5 min. The syringe was left *in situ* for an addition 4 min before being retracted.

The lesion coordinates, in mm, for the ATNx1 group were: anteroposterior, −0.6 relative to bregma; mediolateral, ± 0.9 and ±1.8 from the midline; dorsoventral, −7.0 (medial site) and −6.3 (lateral site) from bregma. For the ATNx2 group, the dorsal–ventral coordinates were set at −7.1 (medial) and −6.4 (lateral) from bregma. For the sham surgeries, the syringe was lowered to +0.2 above the target site for a few seconds, and then removed. No NMDA was injected in these rats. After removal of the Hamilton syringe, the incision was cleaned and sutured. A topical antibiotic powder [Aureomycin; Fort Dodge, Animal Health, Southampton, UK (cohort 1); Dalacin C, clindamycin hydrochloride; Pharmacia Ltd, Kent, UK (cohort 2)] was applied. The rats also received glucose–saline (5 mL s.c.) for fluid replacement, and were then placed in a recovery chamber until they regained consciousness (i.e., movement and righting reflex). Rats were given the analgesic Metacam (0.06 mL s.c.; 5 mg/mL meloxicam; Boehringer Ingelheim Vetmedica, Germany). A respiratory stimulant millophylline (0.1 mL s.c.; Arnolds Veterinary Products, Shropshire, UK), an antimicrobial Baytril in their water (2.5%; Bayer Ltd, Animal Health Division, Ireland), and a low dose of diazepam (0.07 mL s.c., 5 mg/mL; CP Pharmaceuticals Ltd, UK) was administered to facilitate post-operative recovery as needed. All animals were monitored carefully until they had fully recovered.

### Histology

Following behavioural testing, the animals were administered an intraperitoneal injection of a lethal overdose of Euthatal (200 mg/mL sodium pentobarbital, Marial Animal Health Ltd., Harlow, Essex, UK) and perfused intracardially with 0.1 m phosphate-buffered saline (PBS) followed by 4% paraformaldehyde in 0.1 m PBS (PFA). For cohort 2, sodium fluoride was added to the PFA to prevent dephosphorylation as some tissue was collected for additional analysis of CREB and phosphorylated CREB (see Dumont *et al*., 2012). The brains were extracted from the skull and placed on a stirrer to postfix in PFA for 4 h, after which the brains were placed in 25% sucrose overnight. The brains were frozen on a microtome (Leica, UK) and sectioned at 40 μm in the coronal plane. One-in-five sections were mounted and stained with Cresyl Violet, a Nissl stain.

### Volumetric analysis

The extent of the anterior thalamic lesions was estimated in both cohorts (ATNx1, *n *=* *15; ATNx2, *n *=* *14), along with any unintended hippocampal damage. Each lesion outline was mapped onto six corresponding coronal sections taken from a rat brain atlas (Paxinos & Watson, 2005; from bregma −1.08 to −2.28 mm). Likewise, any areas of hippocampal cell loss were plotted onto 20 coronal sections (Paxinos & Watson, 2005) from −1.80 to −6.36 mm with respect to bregma. These images were scanned, and the area of damage was quantified using the programme analySIS^D (Soft-Imaging Systems, Olympus). For this purpose the hippocampus separately comprised the dentate gyrus, CA fields and subiculum.

### Behavioural testing

Cohort 1 had received prior spatial (T-maze alternation) and non-spatial (spontaneous object recognition) testing. The rats were ˜ 12 months old at the start of the present study. Cohort 2 had also received prior T-maze alternation testing but otherwise was experimentally naive, and the rats were ˜ 7 months old.

### Experiment 1: biconditional discriminations (nose poke; cohort 1)

#### Apparatus and room

**Figure 1:**
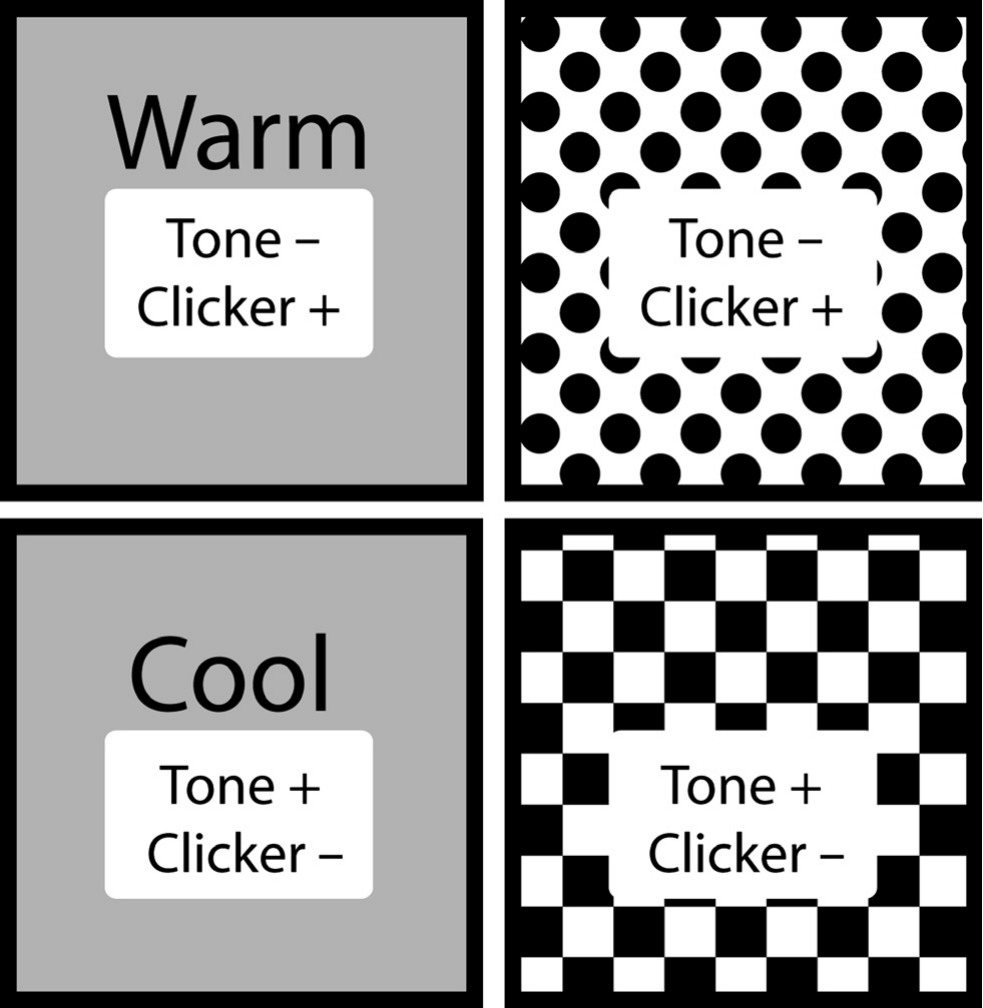
A schematic diagram of the four different contexts inside the operant boxes (two thermal on the left, two visual on the right). In all contexts, tones and clickers are presented; however, only the tone or clicker is paired with food pellets in a given context. The + sign indicates the reinforced stimulus and the – sign indicates the non-reinforced stimulus. Note: warm and cool contexts alternated locations (top; bottom). The visual contexts remained in the same place with the spotted pattern on top and the checkered pattern on the bottom.

Four operant chambers were customised so that each could appear unique (Fig. 1). The four chambers (internal dimensions: 24.5 cm wide × 23 cm deep × 21 cm high; Campden Instruments Ltd., UK) were arranged in a 2 × 2 layout on shelves at the shorter wall of the room (327 × 187 × 254 cm; room A) directly opposite the door. The lowest and highest shelves were 92 and 142 cm above the floor, respectively. Each chamber had three aluminium walls and ceiling; a Perspex door formed the fourth wall. The doors of the sound-attenuating boxes for each of the chambers remained open; therefore, each box received ambient light from a brightly lit room as well as local illumination from a single 15-V, 24-W light situated in the centre of each of the chamber ceilings. A speaker mounted above the ceiling of each of the chambers delivered two auditory stimuli, a 2-Hz tone and a 10-Hz series of clicks at an intensity of ˜ 75 dB(A weighting). On the left chamber wall, a transparent plastic flap (6 cm high × 5 cm wide) blocked the entrance to the food-well where food pellets (45 mg; J. Noyes, Lancaster, NH, USA) could be dispensed. The plastic flap was hinged at the top of the food-well aperture and, if the rats pushed the flap > 2 mm, a microswitch was activated resulting in a single response being recorded.

The two chambers on the left of the 2 × 2 layout were designated ‘thermal’ contexts and the two chambers on the right were the ‘visual’ contexts (Fig. 1). The aluminium walls of the visual contexts were covered with wallpaper protected from the rats by clear Perspex sheets. The top chamber was covered with spotted paper (white background with filled black circles, 1.5 cm in diameter, with a centre-to-centre distance of 2.5 cm), and the bottom chamber was covered with checkered wallpaper (a series of alternating, 3 cm, black and white squares; see Fig. 1). The floor of the visual contexts was constructed from stainless-steel rods. In contrast, the floor of the thermal chambers was aluminium with a bracket fixing allowing two Thermos picnic blocks (model no IP400; 9 cm wide × 3.7 cm deep × 16 cm long) to fit underneath and make contact with the floor of the chamber. For the warm context, the Thermos blocks were first heated in a microwave for 2-3 min. Placing the heated blocks under the floor for 10 min raised the floor temperature to 35°C; this then dropped to 32°C over the course of 30 min. The cool context was created by placing two frozen Thermos blocks below the floor. The temperature of the cool context dropped to 10°C, and then increased to 12°C over the course of 2 h. The heated Thermos blocks were replaced every 30 min, whereas the frozen Thermos blocks were replaced every 2 h (for more details see Ward-Robinson & Honey, 2000). The warm and cool contexts changed location from the top operant chamber to the bottom chamber (and *vice versa*) every 2 days to reduce the possibility of rats using visual cues to solve the biconditional rule (e.g. from observation of the test room through the Perspex door). In contrast, the two visual contexts remained in the same location (i.e., the top chamber was always spotted and the bottom chamber was always checkered).

#### Pre-training

The rats received 2 days of pre-training where they were placed in the operant chambers (without the wallpaper or Thermos blocks, and with conventional steel rod flooring) in order to habituate the animals to the chambers, and to train them to push the flap in order to obtain a food reward. On the first day the flap remained raised, allowing the rats to obtain their food reward without having to push the flap. On the second day the flap was lowered, so the rats had to push the flap to obtain the food rewards. On each day the rats were given 20 pellets (two at a time) on a 60-s variable-time schedule (range 30–90 s).

#### Procedure

Following pre-training, the rats received 20 days of training, with one session per day in each of the four contexts (warm, cool, spotted, checkered). In each context, the tone and the clicks were presented 10 times each in a pseudo-random sequence with the following rule: no more than two trial types (i.e., clicker or tone) occurred in succession. The duration of both auditory stimuli was 10 s, and the presentation of the food reward occurred at the offset of the stimuli. The inter-trial interval (i.e., the time between the offset of one auditory stimulus and the onset of another) was 30 s. When rats were placed in one of the thermal and one of the visual contexts (e.g. cool or checkered), the tone was reinforced (i.e., followed by food reward), whereas the clicker was not (Fig. 1). In contrast, when those same rats were placed in the remaining two contexts (e.g. warm or spotted), the clicker was reinforced and not the tone (Fig. 1). The auditory stimuli that were reinforced in the visual and thermal contexts were fully counterbalanced. The order of presentation of the contexts across the 20 days was also counterbalanced so that every context was presented during the first, second, third and fourth session of each day. Additionally, placement in any one of the contexts was equally likely to be immediately followed or preceded by placement in any of the other three contexts. The behavioural response measured was the number of food-well entries (i.e., the flap covering the food-wells being pushed > 2 mm in order to activate the microswitch) during the 10-s duration of the auditory stimuli as well as the 10 s prior to their onset for baseline recordings. The delivery of the food reward did not depend upon the rats’ behaviour.

### Experiment 2: biconditional learning (open arena digging; cohort 1)

Experiment 2 examined whether rats with lesions to the anterior thalamic nuclei were able to acquire biconditional rules on tasks designed to manipulate distal location cues and proximal context cues jointly, and then these same cue types separately. In contrast to experiment 1, only digging in the correct food cup in the appropriate location or context was rewarded. The first stage (experiment 2A) consisted of a discrimination task to determine whether both groups of rats could distinguish the digging media used in the subsequent biconditional problem. This task was followed by five further stages (experiments 2B–F) that examined performance when the biconditional rule depended on different cue types.

#### Experiment 2A: digging media discrimination

##### Apparatus and room

Animals were tested in either a white opaque plastic test box (40 cm long × 20 cm wide × 12.5 cm high; context 1) or in a blue semi-transparent plastic test box (33 × 26 × 16.5 cm, 16 L; Wham Crystal, Whatmore Creative Plastics, http://www.whamproducts.co.uk; context 2). (Two different test boxes were used as both boxes were required for the biconditional task in experiments 2B and 2C). Regardless of the test box, two digging cups were placed in the middle of each of the shorter walls of the box (22 cm between cups in context 1 and 15 cm between cups in context 2). Each digging cup consisted of a black plastic cylinder with an internal diameter of 7 cm and a height of 6 cm. The base of the cylinder was made of a grey plastic square (9 cm × 9 cm). Velcro secured the cups to the floor to prevent the rats from tipping them over while digging. The two black digging cups were identical during pre-training and contained sawdust. However, during all of the biconditional testing, the two cups and the media inside them differed from each other.

For the discrimination task (experiment 2A), one cup was black and contained shredded red paper whereas the other cup had a white tape surround to produce a checkered pattern and contained multi-coloured plastic beads. The food reward was half of a single Cheerio (Nestle, UK) that was buried in the digging media at a depth of ˜3 cm (i.e., half the cup height). To discourage the rats from using odour-guided cues, a perforated metal grid was placed inside the cup to create a false bottom. Cereal loops were placed under this grid, where they could not be retrieved by the rats. These cereal loops were replaced with fresh ones twice a week. In addition, cereal crumbs were mixed with the digging medium. The pre-training and testing took place in a room (280 cm long × 280 cm wide × 256 cm high; room B) that contained a variety of distal cues (e.g. posters, door, shelves fixed on a wall containing various objects). These distal cues were visible from any corner of the room. The room was illuminated with eight spot bulb lights fixed on the ceiling.

##### Pre-training

Half of the rats in cohort 1 were placed singly in the white opaque plastic test box located on a table (122 cm × 53.4 cm × 70 cm) next to the door of the room half-way along a wall (place 1) whereas the other half was placed in the blue transparent plastic test box located (place 2) on a second table (102 cm × 56 cm × 76 cm) positioned close to a corner, diagonally from place 1. The two tables were 180 cm apart, and the long side of the box was always 20 cm away from the walls. The illumination levels in places 1 and 2 were 151 and 108 lux, respectively. Each test box contained two identical digging cups filled with sawdust. Initially, the food reward was placed on top of the medium and was visible to the rats. Then, as pre-training progressed, the reward was buried deeper and deeper into the sawdust forcing the rats to dig into the medium to retrieve the food. Pre-training lasted between 4 and 6 days, i.e., until each rat dug reliably to retrieve the rewards.

##### Procedure

Five rats were simultaneously brought to the test room in an enclosed carrying box made of aluminium. Each rat was in a separate container and could not see the surrounding environment. The rats were run in spaced trials, i.e., one after the other for each trial. Consequently, there was an inter-trial interval of ˜2–3 min.

**Figure 2:**
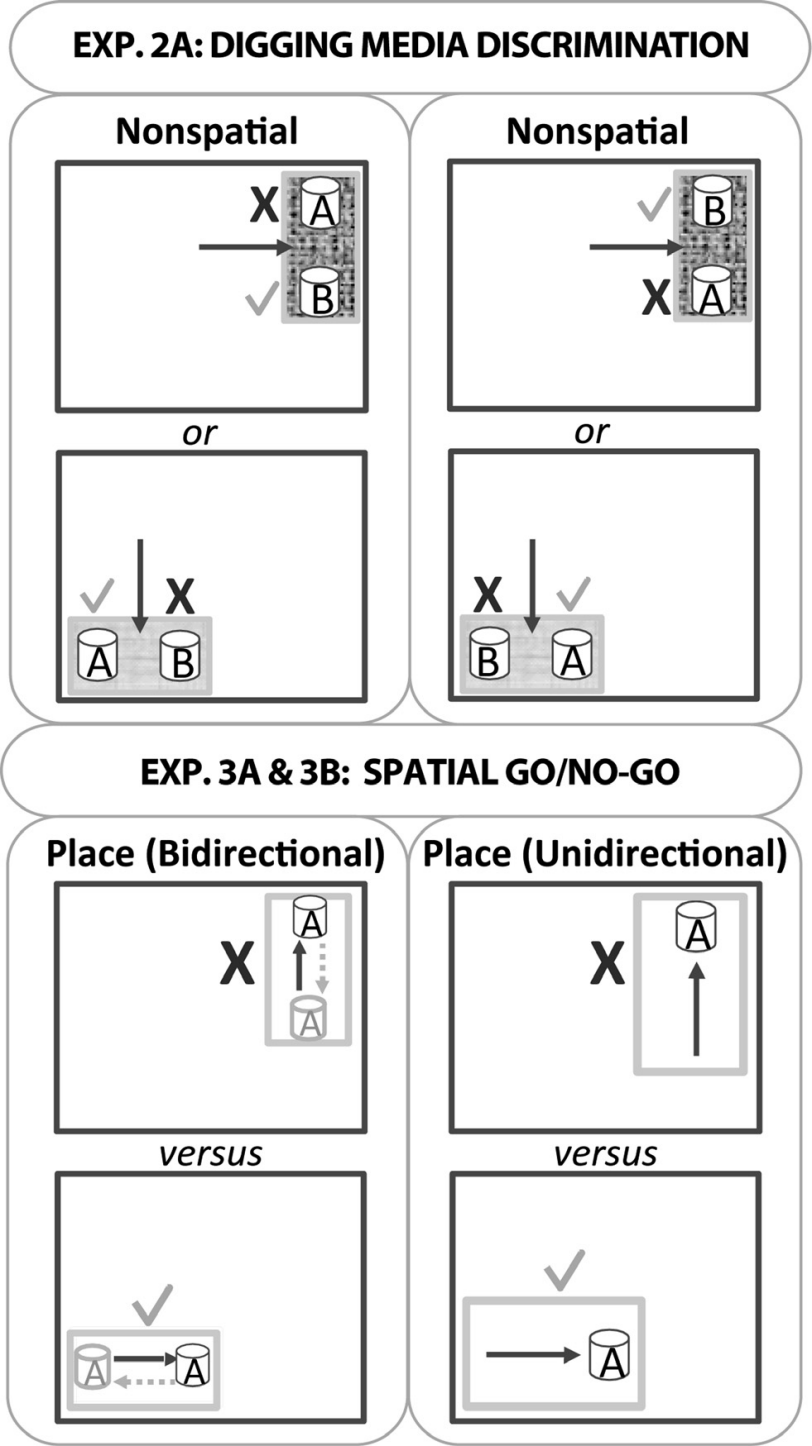
Schematic diagram depicting the nonspatial (experiment 2A) and spatial (experiments 3A and 3B) discrimination tasks. Experiment 2A: this nonspatial discrimination task involved the simultaneous presentation of two different digging media (one always rewarded). The large dark grey outlines represent the testing room, and the smaller grey rectangles represent the plastic test boxes in which the digging cups were placed. The rat was placed in the middle of the test box to start each trial (arrow). For this discrimination rats were tested in one of two different local contexts and one of two locations (these remained constant for a given rat). Consequently, half were tested as shown in the upper pair of drawings and the other half were tested as shown in the lower pair of drawings. The ticks indicate the correct digging cup and the cross indicates the incorrect cup. Experiments 3A and 3B: these go/no-go spatial discriminations involved rewarding the rat for digging in one location (tick) but not in a second location (cross). The black arrow shows the direction the animals ran towards the digging cup(s). In experiment 3A, the dashed grey arrow and grey cup indicate that for half the trials the rats ran towards the digging cup in the opposite direction to the black arrow; therefore, the rats were required to approach the digging cup from two directions in each location (bidirectional). In experiment 3B all trials in a given test box were in the same direction (unidirectional). The diagram is not drawn to scale, nor do the depicted locations represent all test room conditions (see text for the locations of the test boxes in each room). Note that the test box used for experiment 3B was larger than that used for experiment 3A.

Animals received 16 trials per day, for 3 days. Each trial began by placing the rat in the middle of the test box, equidistant from the two digging cups. The rat then explored the cups, one of which contained multi-coloured plastic beads (checkered cup) and the other red shredded paper (black cup). For each rat only one digging medium–cup combination was associated with a reward. For half of the rats this was the beads–checkered cup and for the other half only the paper–black cup combination was rewarded (Fig. 2). The correct digging medium in the white box (context 1 +  place 1) was the multi-coloured beads (i.e., not the shredded paper), whereas the opposite was true in the blue box (context 2 +  place 2). During the digging discrimination, each rat was only tested in one of the two boxes. For this reason, the correct digging medium, the location of the box and the box itself were counterbalanced across the ATNx1 and Sham1 groups. The left and right positions of the correct cup were counterbalanced pseudo-randomly with the following rules: (i) the correct cup occupied the left and right side of the box equally (i.e., eight trials each), and (ii) the correct cup occupied the left or right side for a maximum of three consecutive trials. A correct choice occurred when a rat dug in the correct cup and retrieved the food. Animals were allowed to put their paws on the medium or to smell the medium before making a choice. An incorrect choice was scored when the rat dug in the unbaited cup, resulting in the removal of the correct cup. The rat was left for an extra 5 s before being taken out of the box. At the end of each trial, the rat was returned to the enclosed aluminium carrying box. Behavioural testing was conducted by two experimenters, one of whom remained blind to the group designations throughout the study. For experiments 2 and 3, the details of the procedures remain the same unless otherwise stated.

#### Experiment 2B: context + place biconditional discrimination

Cohort 1 were next trained on a biconditional rule where the choice of cup for food reward was determined by both the appearance of the test box and its location within the room, i.e., both place and local context signalled the correct choice. This experiment used the same apparatus and room as experiment 2A (see Table1).

##### Procedure

**Figure 3:**
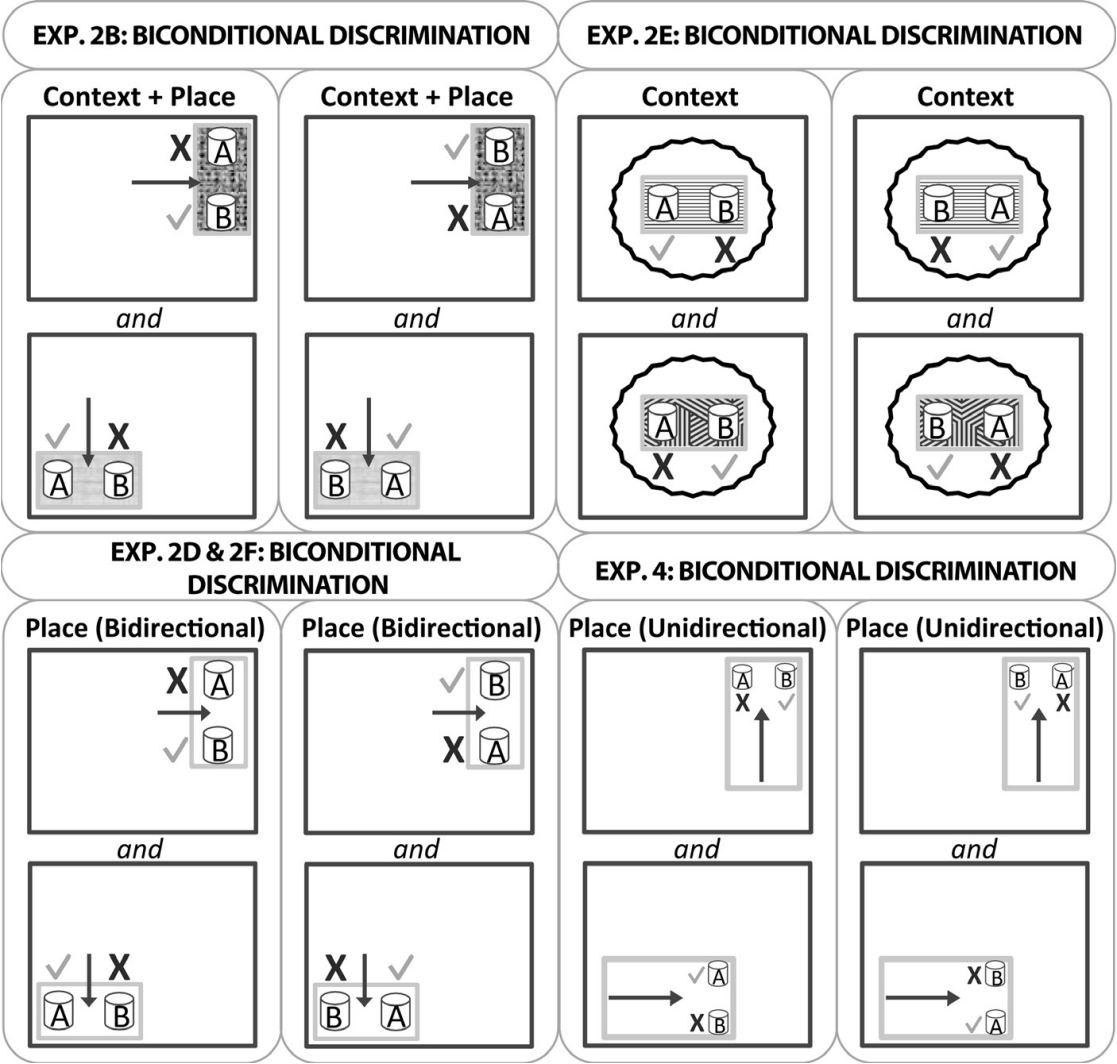
A schematic diagram of the biconditional discriminations in experiments 2 and 4. The dark grey outlines represent the testing room, and the smaller grey rectangles represent the plastic test boxes in which the digging cups were placed. Two different local contexts (test boxes) and locations were used for the context + place biconditional (experiments 2B and 2C). The place biconditional tasks (experiments 2D, 2F and 4) used the same test box for the two locations. In experiments 2D and 2F, the rat was placed between the two digging cups and, therefore, the rat could approach the digging cups going in one of two directions (bidirectional). In contrast, the two digging cups were placed side by side in experiment 4, and the rat always approached the two choice digging cups from a single direction (unidirectional). In the context biconditional task (experiment 2E) the wavy black lines represent the curtain that prevented the rats from seeing the walls while two different local contexts were used (different test boxes). The ticks indicate the correct digging cup while the cross indicates incorrect responses. The black arrow shows where the rat was placed in the test box (experiment 2B) or the direction the animals ran towards the digging cup(s) (experiments 2D, 2F and 4). The diagram is not drawn to scale, nor do the depicted locations represent all test room conditions (see text for the locations of the test boxes in each room). Note that the test box used for experiment 4 was larger than that used for the other experiments (apart from experiment 3B).

Following from the media discrimination task, an additional box and location was introduced on half the trials to help create the biconditional rule (see Fig. 3). For the biconditional problem all of the rats learnt that in the white box (context 1 in place 1) the beads were correct, whereas in the blue box (context 2 in place 2) the shredded paper was correct. The medium that was previously rewarded during the digging media discrimination (experiment 2A) remained the correct medium for the context (and place) in which it was previously correct, but became the incorrect medium in the other context (and place). Consequently, each medium was correct on 50% of the trials. As in experiment 2A, the relative left and right position of the digging cups and the box (context + place) were counterbalanced pseudo-randomly with the one additional restriction, that the correct cup was located equally in both the white and blue boxes, and for no more than three consecutive trials. To eliminate the use of odour cues made by the rats exploring the cups (e.g. marking the cup with urine), the same two cups were used in both the boxes (i.e., for rewarded and non-rewarded trials). The rats received 16 trials per day until the Sham1 rats reached a mean of 80% correct responses for two consecutive days.

#### Experiment 2C: reversal of context + place biconditional discrimination

The reversal task measured whether the rats were primarily attending to the context cues, the place cues, or both. The contingencies associated with the local context cues (floorings) were, therefore, reversed but the biconditional rule remained constant with respect to place (room location). Consequently, a rat that only relied on local context cues should now perform below chance whereas a rat that relied solely on the distal cues (i.e., room location) should remain above chance. For these reasons, the experiment used the same apparatus and room as experiment 2B.

##### Procedure

The procedure was the same as experiment 2B; however, the boxes swapped location so that the white box (context 1) was now in place 2 and the blue box (context 2) was in place 1. The correct cup remained constant with respect to place but not to local context. Therefore, the beads were still correct in place 1, but in the blue box, whereas the shredded paper was still correct when in place 2, but now in the white box. The rats received one session of 16 trials.

#### Experiment 2D: place biconditional discrimination

This experiment examined whether rats with lesions to the anterior thalamic nuclei (cohort 1) were able to acquire a biconditional rule based solely on spatial location. For this reason, local contextual cues became irrelevant.

##### Apparatus and room

The room was the same as experiments 2A–2C. Now, all trials involved the same clear plastic test box (not used before; 40 cm × 30 cm × 12 cm; Smartstore, Sweden) with black opaque handles (20 cm long and 2.5 cm thick) starting 5 cm from the edge of the short walls. The two digging cups were placed along each of the shorter walls (one on each side of the box), and were 22 cm apart.

##### Procedure

The biconditional task was modified (see Fig. 3), so that only room location determined the correct cup in which to dig. Multi-coloured beads (but not shredded paper) were correct in place 1, while shredded paper (but not coloured beads) was correct in place 2. The single test box was moved between the two locations between trials. The location of the box (place 1 or 2) was determined pseudo-randomly (see experiments 2A and 2B). Rats were trained for 16 trials per day until the Sham1 group performed at 80% correct.

**Table 1:** Testing arrangements and outcomes of the various biconditional tasks and place discrimination (Go/No-go)

Experiment	Description	Group	Room	Direction	Impaired?
Exp 1	Biconditional discrimination (nose-poke)	ATNx1	A	n/a	No
Pre-training	Pre-training (digging in cup)	ATNx1	B	n/a	n/a
		ATNx2	C	n/a	n/a
Exp 2A	Digging media discrimination	ATNx1	B	n/a	No
Exp 2B and 2C	Biconditional (open area, digging)	ATNx1	B	Bidirectional	Partial
Exp 2D and 2F	Place biconditional	ATNx1	B	Bidirectional	Yes
Exp 2E	Context biconditional	ATNx1	B	n/a	No
Exp 3A	Go/No-go	ATNx2	B	Bidirectional	Yes
Exp 3B	Go/No-go	ATNx2	D	Unidirectional	Partial
Exp 4	Place biconditional	ATNx2	D	Unidirectional	Yes

Only the lesion groups are displayed in the Table, but the respective control group was also tested in the same room (i.e., ATNx1 with Sham1, ATNx2 with Sham2). Whether the animals were tested approaching the digging cup from a single direction (unidirectional) or two directions (bidirectional) is noted. Performance is indicated as being unimpaired (‘No’), markedly impaired (‘Yes’) or only mildly impaired (‘Partial’).

After completion of the next experiment (2E), the rats were returned to the procedure in experiment 2D for one final session (experiment 2F).

#### Experiment 2E: context biconditional discrimination

This experiment tested whether rats with lesions to the anterior thalamic nuclei could acquire biconditional rules based solely on the local contexts provided by the test boxes.

##### Apparatus

The two distinctive digging cups (15 cm apart) were placed in one of two semi-transparent plastic boxes (both 33 × 26 × 16.5 cm; Wham Crystal, Whatmore Creative Plastics). The two boxes could readily be distinguished as one box had laminated wall panels composed of white and red triangles and a green textured Duplo (Lego, UK) base covering the floor (context 1). The second box had a smooth, checked (black and white) laminated floor but plain walls (context 2). The two boxes could be placed on a table in the centre of the test room. Black curtains were placed around the table and box, preventing the use of distal cues (see Fig. 3).

##### Procedure

The procedure was the same as experiment 2B, except that now only local context cues solved the biconditional problem. Consequently, the coloured beads were correct in the Duplo base box (context 1), whereas the shredded paper was correct in the checkered floor box (context 2). The same two distinctive cups were used in both boxes.

#### Experiment 2F: place biconditional discrimination

To determine whether there might have been some functional recovery, the place biconditional task (experiment 2D) was repeated for one session only.

### Experiment 3: spatial go/no-go discrimination (cohort 2)

The selective deficit found for the biconditional discrimination that taxed the use of distal room cues (experiments 2D and 2F; see Results) raises the question of whether the ATNx1 rats could effectively discriminate the spatial cues. This possibility was examined by training a new cohort of rats (cohort 2) to discriminate different spatial room cues.

#### Experiment 3A: spatial go/no-go discrimination (bidirectional)

In experiment 3A the two locations to be discriminated were each approached from two directions (‘bidirectional’), to match the test arrangement used in experiment 2. This testing arrangement can be contrasted with ‘unidirectional’, in which the rat would only approach a given digging cup from a single direction.

##### Apparatus and room

Cohort 2 was tested in the same white plastic test box (40 cm long × 20 cm wide × 12.5 cm high) as was previously used in experiments 2A–2C. A single cup filled with sawdust was presented in the centre of the side with a short wall (Fig.2). This cup was identical to that used in the pre-training stage of experiment 2A. The food reward was half a loop of a single Cheerio (Nestle, UK) that was buried in the digging media and the same precautions as described above were used to stop the rats from solving the task by locating the food reward by its scent.

Pre-training took place in a narrow room (330 cm long × 190 cm wide × 256 cm high; room C). Visual cues such as posters and shelves were fixed on the walls. A table was placed near the back wall of the room. Testing then occurred in a different room (room B; 280 cm long × 280 cm wide × 256 cm high) that also contained a variety of distal cues (e.g. posters, door, shelves fixed on a wall containing various objects). Room B was the same as that used for experiment 2. Because there were no large objects in the centre of the room, the distal wall cues were visible from any room corner. The room was illuminated with eight spot bulb lights fixed on the ceiling, and the mean luminance in place 1 was 123 lux and that in place 2 was 124 lux.

##### Procedure

Initial pre-training was identical to that described for experiment 2 except that only one open test box was used throughout pre-training. As in experiment 2, four or five rats were simultaneously brought to the test room in an enclosed carrying box made of aluminium and run in spaced trials with an inter-trial interval of 2–3 min.

A single cup filled with sawdust was placed along one of the short walls in the white plastic box on each trial. The test box was always placed in one of two table-top locations in room B. For any given rat, the cup was always baited in one room location (go response), but never baited when placed in the other room location (no-go response; Fig. 2), regardless of the direction from which the rat approached the cups. As a result, on each trial a single digging cup could be found in four places: (i) north end of the box in the go location; (ii) south end of the box in the go location; (iii) north end of the box in the no-go location; and (iv) south end of the box in the no-go location. The distance between the two places the cup could be found within the testing box was 22 cm, whereas the go and no-go locations were ˜130 cm apart. One table (122 × 53.4 × 70 cm) was located near the door, whereas the second table (102 × 56 × 76 cm) was placed within a corner (i.e., two adjacent walls). At the start of each trial, the rat was placed at the end of the box furthest away from the digging cup. Learning was assessed by comparing the latency of the rat to dig when the box was in the baited location and the latency to dig when the box was in the never-baited position. Each trial had a time limit of 20 s, after which the rat was removed. If the rat dug in the correct location it was removed as soon as it had consumed the cereal reward, but if the rat dug in the incorrect location it was left for an extra 5 s before being removed from the box. The trial order was counterbalanced pseudo-randomly between the two locations (correct and incorrect; see experiments 2A and 2B). In addition, the direction in which the animal ran to the digging cup (i.e., the start location was either to the north or to the south end of the box) was also counterbalanced across the 16 trials pseudo-randomly with the following rules: (i) the rat ran to the cup from both directions equally (i.e., eight trials each); and (ii) the rat ran towards the digging cup in the same direction for a maximum of three consecutive trials.

#### Experiment 3B: spatial go/no-go discrimination (unidirectional)

This experiment also examined acquisition of a spatial go/no-go task but now each cup was approached from just one direction, i.e., a constant direction (‘unidirectional’).

##### Apparatus and room

Cohort 2 were tested in a transparent box (52 cm long × 33 cm wide × 17 cm high, 45 L; Crystal, Whatmore Creative Plastics) that was larger than the ones used in experiments 2 and 3A. (The enlarged box was required for the subsequent biconditional protocol in experiment 4.) The rats were tested in a new room (room D, 300 cm long × 275 cm wide 239 cm high; see Fig. 2 and Table1). The plastic digging cup was placed in the centre of the short wall of the rectangular box.

The test box was placed in one of two distinctive room locations. Place 1 was by the middle of a shelving unit made of metal bars (52 cm between shelves). The box was 89 cm above the floor and 6 cm from the wall closest to the side of the box. The wall in front of the box was 69 cm away while the wall to the rear was 154 cm away. Place 2 was in the diametrically opposite corner of the room on top of a trolley (71 cm high). The wall by the side of the box was 11 cm away. The wall to the rear of the box was 26 cm away. The centres of place 1 and place 2 were ˜ 150 cm apart. The room was illuminated with eight small light bulbs, with additional lighting near place 1 to match luminance levels. The illumination levels were 263 lux (place 1) and 262 lux (place 2).

##### Procedure

The procedure was exactly the same as experiment 3A except that the rats only ran towards the digging cup from a single direction (i.e., unidirectional). From the experimenter’s point of view the rats always ran from left to right, with the wall closest to animal’s left flank irrespective of location.

### Experiment 4: Place biconditional disrimination (unidirectional; cohort 2)

This experiment examined whether having learnt to discriminate two locations (experiment 3B) enabled the rats to solve a biconditional problem that involved the same spatial cues. As in experiment 3B, the two choice cups were approached from a single direction (i.e., unidirectional; Fig. 3).

#### Apparatus and room

The test room (D) and box were the same as experiment 3B (Table1). Two digging cups (a black cup filled with red shredded paper and a checkered cup containing beads) were placed along the short wall of the rectangular plastic box with a 15-cm gap between the cups. The same box was used in both locations.

#### Procedure

The procedure was the same as experiment 2D (place biconditional learning) except that the animals were always released facing the cups, and so always approached them from the same direction for a given test box location (Fig. 3).

## Results

### Histology

**Figure 4:**
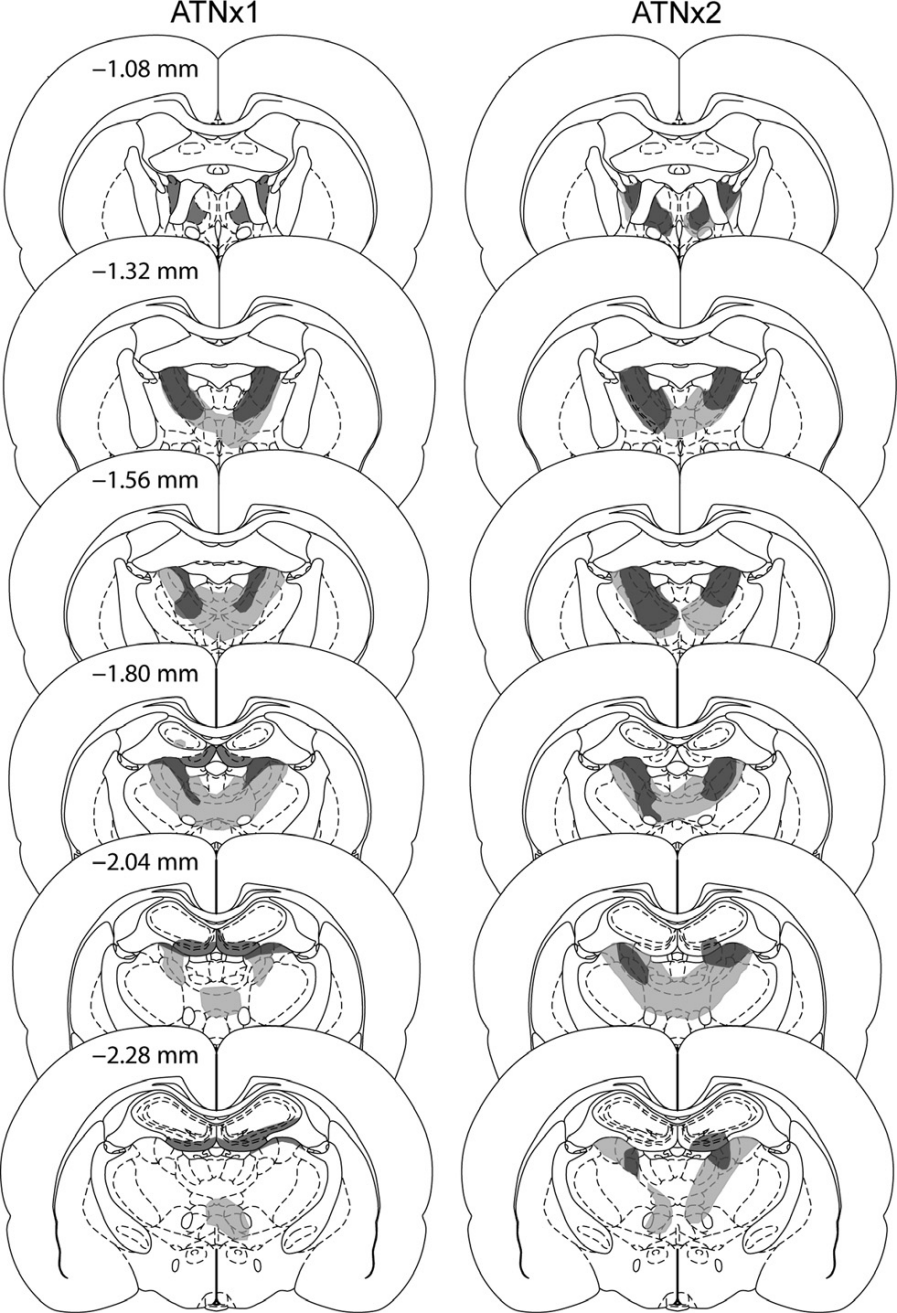
The minimum (dark grey) and maximum (light grey) extent of the lesions for the ATNx1 and ATNx2 groups. The numbers refer to the approximate distance of the section in mm caudal to bregma. The sections are modified from Paxinos & Watson (2005). It should be noted that other rats in the ATNx1 and the ATNx2 groups had less unintended hippocampal damage than that depicted.

Figure 4 shows the individual cases with the minimum and maximum cell loss in the ATNx1 and ATNx2 groups. Those animals with anterior thalamic damage that involved < 50% of the total structure were excluded from the behavioural analyses.

#### Cohort 1

Three ATNx1 animals were excluded as > 50% of the ATN was spared. For the remaining 12 ATNx1 rats the total area of cell loss in the anterior thalamic nuclei was between 52 and 94% (mean, 76%; median, 76%). Any sparing typically occurred within the caudal anterior thalamic nuclei, often in the most ventral portion of the anterior medial nucleus. However, two rats exhibited the opposite pattern with a more complete lesion at the caudal end of the anterior thalamic nuclei, with sparing occurring rostrally. These two animals had some sparing to the anterior dorsal nucleus. In 11 out of 12 cases, there was partial damage to the rostral and dorsal portions of the laterodorsal nucleus, which in three cases was unilateral. In those rats with larger lesions, there was also some restricted damage to the parataenial nucleus (*n *=* *7; unilateral in two cases), the paraventricular nucleus of the thalamus (*n *=* *3), the reticular nucleus (*n *=* *6; unilateral in three cases) and nucleus reuniens (*n *=* *7).

In all cases there was some isolated cell loss in the hippocampus. Most of this hippocampal damage was restricted to the very rostral (septal) part of the ventral (inferior) blade of the dentate gyrus. Of the twelve cases, eleven had restricted bilateral damage to just this part of the septal dentate gyrus while in one case it was unilateral. In some cases this cell loss extended into the immediately adjacent CA3 (*n *=* *9, of which three had unilateral cell loss). It is important to stress that this hippocampal damage was very limited, producing a mean loss of 3.3% of the total hippocampus (range 0.2–5.8%). Given that the majority of the rats had some restricted cell loss to the dentate gyrus, the percentage loss to this region was examined separately. A mean of 8.0% loss (range 0.8–14.6%) of the dentate gyrus was found. In one case the injection tracts in the fornix appeared to induce some additional damage (primarily unilateral), whereas in three other cases the fornix appeared intact but somewhat distorted in both hemispheres.

#### Cohort 2

Four ATNx2 rats were excluded from further analysis. In three cases, there was excessive sparing of the anterior thalamus and in one further case the lesion extended into the medial septal nuclei. In the remaining 10 cases there was considerable cell loss in the anterior thalamic nuclei with the lesion occupying 73–100% (mean, 93%; median, 96%) of the area. In the cases with the smaller lesions, sparing typically occurred in the anterior medial nucleus, and in the right hemisphere. In all 10 cases, the lesion extended posteriorly into the most rostral and dorsal portions of the laterodorsal nucleus and in five cases the lesions reached the rostral cap of the medial dorsal nucleus of the thalamus (only unilateral in three cases). In some cases there was also partial damage to the parataenial nucleus (*n *=* *8), the paraventricular nucleus (*n *=* *5), the reticular nucleus (*n *=* *8, unilateral in two cases), nucleus reuniens (*n *=* *9) and the ventral anterior thalamic nucleus (*n *=* *9, unilateral in four cases).

In five cases there was some restricted bilateral cell loss in the hippocampus; three other rats had restricted unilateral damage to this region. The cell loss was typically confined to the most rostral part of the ventral (inferior) blade of the dentate gyrus (*n *=* *8, in three cases the damage being unilateral), but occasionally the atrophy extended into the immediately adjacent part of CA3 (unilateral, *n *=* *2; bilateral, *n *=* *1). In three cases, the damage reached the medial part of septal CA1 (unilateral, *n *=* *2; bilateral, *n *=* *1). A mean of 1.5% of the total hippocampus was damaged (range 0–5.8%). The percentage loss to the dentate gyrus was also examined separately, and ranged from 0% to 12.8%. A mean of 4.2% (median, 3.0%) loss of the dentate gyrus was found. In one case there was unilateral distortion of the fornix.

### Behavioural testing

Several different behavioural measures were obtained, and as a result the data were analysed differently. In experiment 1, the data were examined by observing the rate of responding (magazine entries) during the presentation of the reinforced stimuli and non-reinforced stimuli. In addition, the data were analysed as percentage ratios [100 × reinforced stimuli/(reinforced + non-reinforced stimuli)]. The percentage ratio scores have the advantage of better controlling for individual variability in responding (e.g. a rat that has overall lower response rates regardless of the condition). For experiments 2 and 4, the rats were presented with a forced-choice response (i.e., one of the two digging cups was correct). The total number of correct trials (out of 16) for the test day was expressed as percentage correct responses. However, in experiment 3 the behaviour measured was latency to dig (maximum 20 s). These data were examined first using latency to dig in the go location compared with the latency in the no-go location, and then as a latency ratio [100 × no-go/(go + no-go)]. While the raw latency scores provide information on the actual time it took the rats to dig in the cup, the percentage ratio scores better compensate for individual variability in performance (e.g. rats that respond faster in both the go and no-go locations).

#### Experiment 1: biconditional discriminations (nose poke; cohort 1)

**Figure 5:**
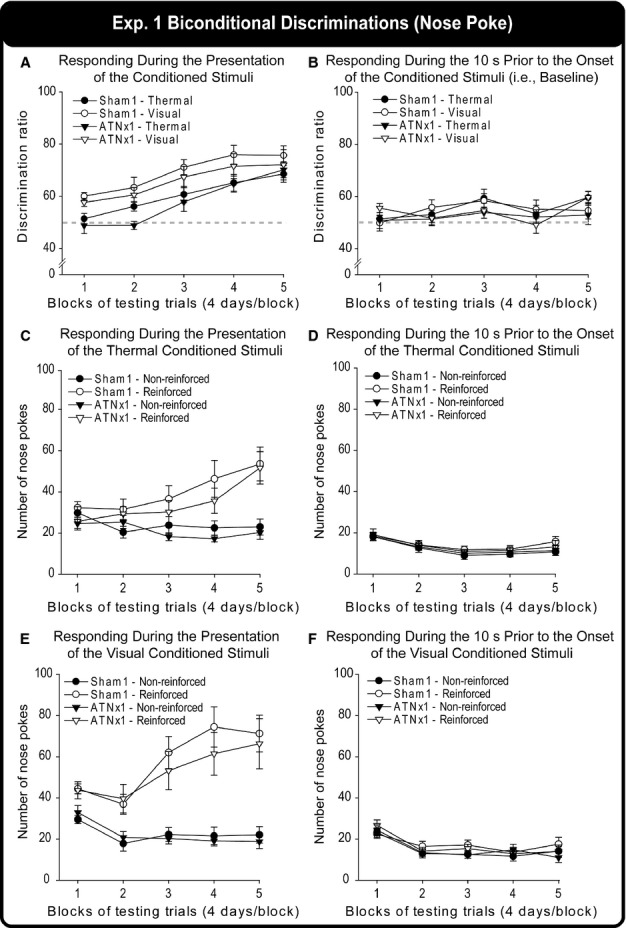
The discrimination ratios of the Sham1 and ATNx1 groups for the thermal and visual contexts across successive blocks of testing trials, (A) during the presentation of the auditory (tones, clickers) stimuli, i.e., during task acquisition, and (B) during the 10 s prior to the onset of these auditory stimuli, i.e., baseline response levels. (Positive scores reflect acquisition of the biconditional task.) Performance on the same task is also shown by separately depicting the numbers of nose pokes for the reinforced and non-reinforced auditory stimuli during their 10-s presentation trials in (C) the thermal and (E) the visual contexts. The numbers of nose pokes in the 10 s preceding the onset of the reinforced and non-reinforced stimuli in (D) the thermal and (F) visual contexts across testing. Note: grey dashed line indicates chance (50%). The graphs show the mean ± SEM scores.

Both groups acquired the tasks, and there was no evidence of a lesion-induced deficit. Figure 5(A) shows the discrimination ratios during the presentation of the auditory stimuli for the thermal and visual contextual biconditional discriminations. The ratios correspond to the number of magazine entries during the 10-s presentation of the correct auditory stimulus in a given context divided by the total number of magazine entries during the presentation of both auditory stimuli for 10 s. The ratios were then multiplied by 100. Therefore, a score of 50 indicates chance performance, i.e., responding to both auditory stimuli equally. A three-way mixed-model anova (Group × Condition × Block) gave a significant main effect of Block (*F*_4,80_* *=* *22.3, *P *<* *0.001), indicating that the discrimination ratios increased over testing blocks (i.e., the performance of the rats improved). There was, however, no significant main effect of Group (*P *>* *0.1). Both the Sham1 and the ATNx1 groups also performed significantly better on the visual contextual discriminations than on the thermal ones (*F*_1,20_* *=* *33.8, *P *<* *0.001). None of the interactions was significant (all *P *>* *0.1).

When the response rates (i.e., number of magazine entries) during the 10-s presentation of the auditory stimuli in the thermal (Fig. 5C) and visual (Fig. 5E) contexts were analysed, an anova also failed to find a significant main effect of Group (*P *>* *0.1). However, similar to the percentage ratio scores, there was a significant main effect of Block (*F*_4,80_* *=* *6.27, *P *<* *0.001) and Condition (*F*_1,20_* *=* *23.8, *P *<* *0.001), showing that the rats performance improved over time and that performance was better in the visual than in the thermal condition. In addition, there was a significant main effect of Reinforcement (reinforced stimuli compared with non-reinforced stimuli), indicating that both groups responded more to the reinforced auditory stimuli than to the non-reinforced auditory stimuli (*F*_1,20_* *=* *57.7, *P *<* *0.001). There was also a significant Condition × Block interaction (*F*_4,80_* *=* *5.92, *P *<* *0.001), Reinforcement × Block interaction (*F*_4,80_* *=* *22.8, *P *<* *0.001), Reinforcement × Condition interaction (*F*_1,20_* *=* *41.4, *P *<* *0.001), and a Condition × Reinforcement × Block interaction (*F*_4,80_* *=* *6.53, *P *<* *0.001). None of the other interactions were significant (all *P *>* *0.1).

Figure 5(B) displays the discrimination ratios during the 10 s prior to the onset of the auditory stimuli for the thermal and visual context of the ATNx1 and Sham1 groups. A three-way anova with the between-subjects factor Group and within-subject factors Context and Blocks just failed to find a significant main effect of Block (*F*_4,80_* *=* *2.5, *P *=* *0.051), indicating that the performance of the rats varied across testing blocks. None of the other main effects or interactions was significant (all *P *>* *0.1). The results indicate that the groups did not differ on baseline (i.e. before presentation of the auditory stimuli) magazine entries in either the thermal or visual contexts.

In addition to the ratio scores, the response rates (number of magazine entries) during the 10 s prior to the auditory stimuli in the thermal (Fig. 5D) and visual (Fig. 5F) contexts were also examined to assess any differences in baseline responding between the Sham1 and ATNx1 groups. There was neither a significant group difference nor any factor × Group interactions (all *P *>* *0.1). Similarly, when responding during the first 10 s prior to the onset of the first auditory stimuli of each day was considered, the groups did not differ from one another (*P *>* *0.1).

#### Experiment 2: biconditional learning (open arena digging; cohort 1)

##### Experiment 2A: digging media discrimination

**Figure 6:**
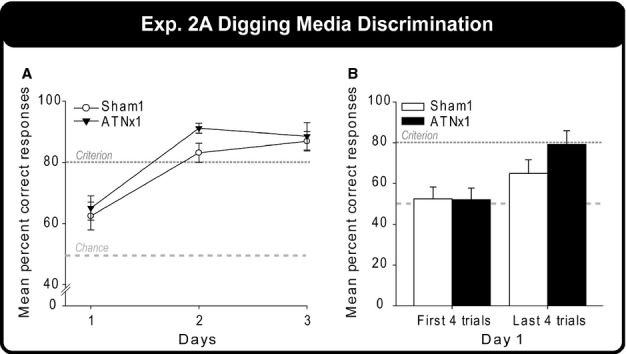
Digging media discrimination task. The graph shows the mean percentage correct responses of the Sham1 and ATNx1 groups for each of the three test days. The light grey long-dashed line indicates chance (50%) and the dark grey short-dashed line indicates criterion (80%). The graphs show the mean ± SEM scores.

Both groups of rats rapidly learnt to dig in just one of the two cups containing different media (beads or shredded paper) for a food reward (Fig. 6). By the second day of testing, both groups were > 80% correct. This acquisition was reflected in a significant main effect of Day (*F*_2,40_* *=* *33.9, *P *<* *0.001), but there was no evidence of a lesion effect (Group, Group × Day, both *P *>* *0.1). Although both groups were above chance by the end of day 1, this reflected the rapid within-session learning, e.g. for both groups the first four trials were at chance (52% ATNx1, 53% Sham1; see Fig. 6B).

##### Experiment 2B: context + place biconditional discrimination

**Figure 7:**
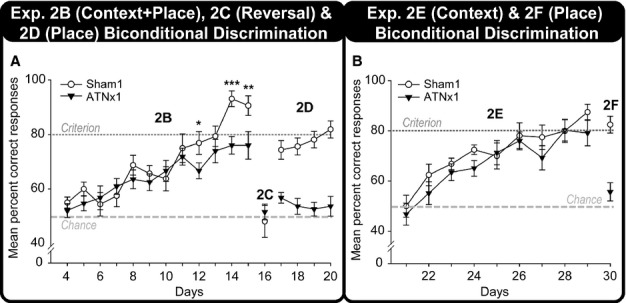
Biconditional discrimination performance. The mean percentage correct responses of the Sham1 and ATNx1 groups across testing days during (A) the context + place biconditional discrimination (experiment 2B), the reversal of the biconditional contingencies (experiment 2C) and the place biconditional discrimination (experiment 2D and 2F), and (B) the context biconditional discrimination (experiment 2E). Note: 2B, experiment 2B (context + place biconditional); 2C, experiment 2C (Reversal), where the context (boxes) were swapped creating incongruent context-place information; 2D, experiment 2D (place biconditional); 2E, experiment 2E (context biconditional); 2F, experiment 2F, where experiment 2D (place biconditional) was repeated for 1 day only; light grey long-dashed line indicates chance (50%) and the dark grey short-dashed line is criterion (80%); *P <* *0.05, **P <* *0.01, ***P <* *0.001. The graphs show the mean ± SEM scores.

The correct choice was guided both by room location and by local test box cues (i.e., cup A was reinforced in box context 1 +  place 1 and cup B was reinforced in box context 2 +  place 2). The total correct scores (out of 16), expressed as percentage correct responses, were compared across days. Both groups steadily acquired this initial biconditional task (Fig. 7A) although the final performance levels of the Sham1 rats were superior to those of the ATNx1 rats. A two-way mixed-model anova (Group × Day) confirmed the gradual improvement in performance (effect of Day, *F*_11,220_* *=* *19.4, *P *<* *0.001) as well as final differences in performance (Group × Day interaction, *F*_11,220_* *=* *1.87, *P *=* *0.045). The simple effects indicated that the Sham1 group outperformed the ATNx1 group on days 12 (*F*_1,240_* *=* *3.91, *P *=* *0.049), 14 (*F*_1,240_* *=* *11.0, *P *=* *0.001) and 15 (*F*_1,240_* *=* *7.99, *P *=* *0.005). The main effect of group was close to significant (*F*_1,20_* *=* *4.01, *P *=* *0.059), and both groups clearly mastered the problem, albeit to different performance levels.

##### Experiment 2C: reversal of context + place biconditional discrimination

For one session only, the two test boxes (local context) swapped locations, i.e., the test box for place 1 (context 1) was now in place 2, and the second test box (context 2) was now in place 1. The reinforcement rule followed the room locations and not the local context cues. Neither the Sham1 nor the ATNx1 group differed significantly from chance performance (Fig. 7A), and the two groups did not differ from one another (all *P *>* *0.1).

##### Experiment 2D: place biconditional discrimination

Only one test box was used throughout this task, and so local context cues were removed, i.e., rats should now rely on distal location information. Consequently, cup A was correct in place 1 while cup B was correct in place 2. The Sham1 group showed a very clear positive transfer effect from experiment 2B (Fig. 7A) as their performance was close to the criterion level from the very first test day. In contrast, the ATNx1 rats performed close to chance. A two-way mixed-model anova (Group × Day) underlined this group difference (*F*_1,20_* *=* *70.5, *P *<* *0.001). There was no effect of Day and no Group × Day interaction (both *P *>* *0.1).

##### Experiment 2E: context biconditional discrimination

In the complementary design to the previous task, the biconditional rule could now be solved by reference to the local context cues (the two distinctive test boxes) but not by using distal room cues (one place was used throughout, with a curtain to block distal cues). It can be seen from Fig. 7(B) that both groups could solve this context biconditional task and there was no evidence of a lesion effect (main effect of Day, *F*_8,160_ =18.4, *P *<* *0.001; effect of Group, *P *>* *0.1) as both groups reached the criterion score level.

##### Experiment 2F: repeat of place biconditional discrimination

The deficit in the ATNx1 rats was reinstated when given one final test session of the place biconditional task (Fig. 7B). While the ATNx1 group dropped to near-chance levels (mean 55% correct), the Sham1 group performed above the 80% criterion level (Fig. 7B). The group difference (*t*_20_* *=* *5.33, *P *<* *0.001) reflected the impairment in the ATNx1 rats.

#### Experiment 3: spatial go/no-go discrimination (cohort 2)

In order to determine whether the anterior thalamic lesions had caused an underlying deficit in distinguishing spatial locations, cohort 2 was rewarded for digging in one room location but not the other (go/no-go).

##### Experiment 3A: spatial go/no-go discrimination (bidirectional)

**Figure 8:**
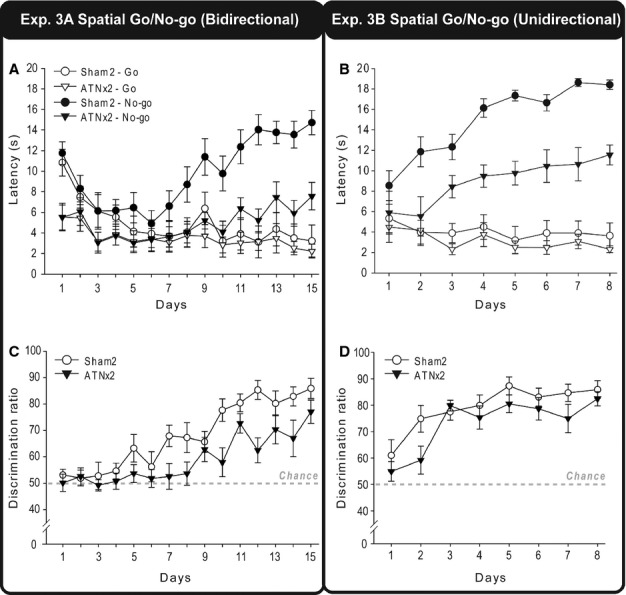
Place discrimination (Go/No-go). The mean latencies (s) during go and no-go trials, and the discrimination ratio [100 × (no-go/(go + no-go)] of the ATNx2 and the Sham2 groups across blocks of testing during the bidirectional go/no-go spatial discrimination task (A and C, experiment 3A) and the unidirectional go/no-go spatial discrimination task (B and D, experiment 3B). Data shown are group means, and the vertical bars are SEM. For the discrimination ratios, a score of 50 represents chance (i.e., equal latencies during both go and no-go trials). Bidirectional, the rat can approach the digging cup from two directions; Unidirectional, the rat always approaches the digging cup from one direction.

When both locations could be approached from two directions (Fig. 2), the Sham2 rats showed much greater differential response latencies on the go and no-go trials with training than did the ATNx2 rats (Fig. 8A). This lesion effect is reflected in an overall group difference in response latencies (lower in the ATNx2 rats; *F*_1,20_* *=* *5.28, *P *=* *0.033) but, more importantly, by the significant Group × Condition (go/no-go) interaction (*F*_1,20_* *=* *20.4, *P *<* *0.001), the Day × Condition interaction (*F*_14,280_* *=* *17.4, *P *<* *0.001), and a three-way interaction between Group, Condition (go/no-go) and Day (*F*_14,280_* *=* *3.71, *P *=* *0.002), indicating that as testing progressed the Sham2 group were able to withhold responding in the no-go location relatively more than the ATNx2 group.

These lesion effects can also be seen when the data are represented as ratio scores [100 × no-go/(go + no-go); Fig. 8C]. There was a significant effect of Group (*F*_1,20_* *=* *9.57, *P *=* *0.006) and Day (*F*_14,280_* *=* *15.0, *P *<* *0.001) The Group × Day interaction approached significance (*F*_14,280_* *=* *1.58, *P *=* *0.08).

##### Experiment 3B: spatial go/no-go discrimination (unidirectional)

Inspection of the latencies to dig suggests that the ATNx2 group remained impaired when trained on a spatial go/no-go discrimination where the rats approached each digging cup from a single direction (Fig. 8B). Comparisons of the latencies on the go and no-go trials gave a main effect of Group (*F*_1,20_* *=* *10.6, *P *=* *0.004) as the ATNx2 rats were faster overall. Both the effect of test Condition, (*F*_1,20_* *=* *184.6, *P *<* *0.001) and the Condition × Day interaction (*F*_7,140_* *=* *21.1, *P *<* *0.001) reflected acquisition of the place discrimination. The Group × Condition interaction (*F*_1,20_ = 17.2, *P *=* *0.001) reflected the Sham2 group’s greater ability to withhold responding in the no-go location compared with the ATNx2 group, i.e., their superior learning. All other interactions were non-significant (*P *>* *0.1).

In contrast, when the data were analysed as ratio scores (Fig. 8D), there was no significant main effect of Group (*F*_1,20_* *=* *2.31, *P *>* *0.1). There was, however, a significant main effect of Test Days, reflecting the improved discrimination between the two spatial locations by both the ATNx2 and the Sham2 groups (*F*_7,140_* *=* *16.6, *P *<* *0.001). The Group × Day interaction was not significant (*P *>* *0.1). In consequence it can be seen that both groups did acquire the place discrimination.

#### Experiment 4: place biconditional disrimination (unidirectional; cohort 2)

**Figure 9:**
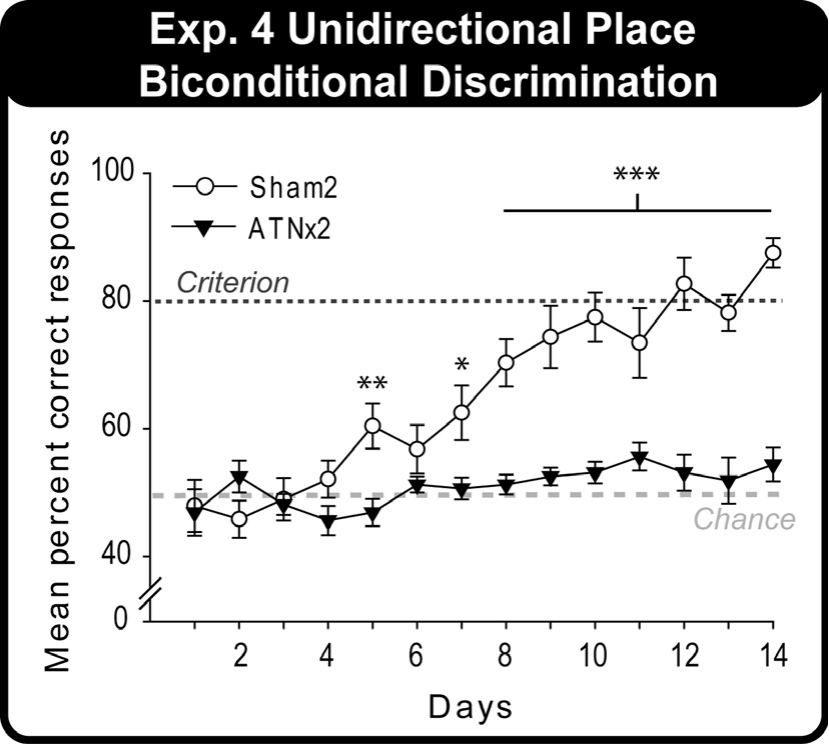
The mean percentage correct responses of the Sham2 and ATNx2 groups across test days for the unidirectional place biconditional discrimination (experiment 4). Data shown are group means, and the vertical bars are SEM. Note: light grey long-dashed line indicates chance (50%) and dark grey short-dashed line is criterion (80%). Significant group differences: *P <* *0.05, **P <* *0.01, ***P <* *0.001.

The ATNx2 group failed to learn this place biconditional task, in contrast to the Sham2 group, which reached the 80% criterion (Fig. 9). This description is confirmed by both the main effect of Group (*F*_1,20_* *=* *34.5, *P *<* *0.001) and the significant Group × Day interaction (*F*_13,260_* *=* *8.40, *P *<* *0.001). The simple effects indicate that the groups differed on days 5 (*F*_1,280_* *=* *8.33, *P *=* *0.004) and 7–14 (Day 7, *F*_1,280_* *=* *6.41, *P *=* *0.012; Days 8–14, *P *<* *0.001), reflecting the significantly improved performance of the Sham2 rats over test days (*F*_13,260_* *=* *22.6, *P *<* *0.001). In contrast, the ATNx2 rats did not improve over testing days (p > 0.1).

### Performance–lesion correlations

In light of the rationale for these studies the potential impact of any unintended hippocampal damage upon the thalamic lesions should be considered. Such damage was always extremely restricted and no significant correlations were found between total extent of hippocampal damage and performance (mean of last two sessions) on those tasks that were disrupted by thalamic surgery (all *P *>* *0.1 for cohort 1 and cohort 2). Likewise the extent of dentate gyrus loss alone did not correlate significantly with any of the behavioural measures impaired by the surgeries (all *P *>* *0.1). Similar analyses found that greater anterior thalamic damage was associated with poorer performance on experiment 4 (*P *<* *0.05, one-tailed), though none of the other correlations was significant.

## Discussion

Rats with lesions of the anterior thalamic nuclei were trained on a series of biconditional discriminations. These discriminations revealed a contrasting pattern of spared and impaired learning. Whether the biconditional task required the rats to approach a covered magazine following food delivery in a chamber (experiment 1) or to dig within cups containing different media (experiment 2E), the surgery did not appear to affect task acquisition when the appropriate choice behaviour was signalled by local contextual cues. That is, rats with anterior thalamic lesions could learn the biconditional rule ‘if A do Y, but if B do X’ when signalled by cues such as hot versus cold floors, dotted versus checkerboard walls (experiment 1), or by dark versus transparent walls and floors (experiment 2E). In contrast, the same surgeries prevented biconditional learning when the conditional cues consisted of different room locations, i.e., if place A do Y, if place B do X (experiments 2D, 2F and 4). For this same reason, the mild deficit in the anterior thalamic lesion group in experiment 2B (Fig.7A) presumably reflected their ability to use local context cues, but not distal spatial cues, when both were present to help solve the same biconditional problem.

For these biconditional experiments, the conditional response comprised either nose-poking (experiment 1) or digging in different media (experiments 2, 3 and 4) for food. These particular behaviours were selected as there was no prior evidence that these responses should be affected by the anterior thalamic lesions. This assumption was borne out by the rats’ intact performance on the contextual nose-poking biconditional task (experiment 1) and by the normal acquisition of the digging media discrimination (experiment 2A; see also experiment 2E). Consequently, the critical feature in determining whether the anterior thalamic lesions disrupted biconditional learning in these experiments appears to be the nature of the conditional signal.

A number of previous studies have explored the impact of anterior thalamic lesions on biconditional learning, with varying outcomes. Based on the present findings it would seem appropriate to divide these previous studies into two categories. The first category comprises conditional stimuli or responses that are not defined by reference to distal spatial cues, e.g. that entail the use of local context cues, auditory signals or egocentric-based responding. The prediction is that anterior thalamic lesions will spare biconditional tasks confined to these elements. This prediction builds on the knowledge that anterior thalamic lesions typically spare the discrimination of simple, elemental stimuli and do not impair egocentric spatial tasks (Aggleton *et al*., 1996, 2009; Warburton *et al*., 1997; Sziklas & Petrides, 1999; Mitchell & Dalrymple-Alford, 2006; Wolff *et al*., 2008). The second category comprises biconditional tasks where the signal stimuli or the conditional responses are distinguished by location cues. This grouping is underpinned by evidence that anterior thalamic lesions impair a range of tasks thought to rely on distal spatial cues (Sutherland & Rodriguez, 1989; Aggleton *et al*., 1995; Byatt & Dalrymple-Alford, 1996; Warburton & Aggleton, 1999; Wilton *et al*., 2001; van Groen *et al*., 2002; Loukavenko *et al*., 2007).

Examples of the first category include when an animal forms a biconditional association between an item and a left–right position that can be defined egocentrically (Chudasama *et al*., 2001; Ridley *et al*., 2002). For instance, when presented with one of two visual stimuli on a screen, rats with anterior thalamic lesions learnt to nose-poke to the left or to the right according to the particular stimulus (Chudasama *et al*., 2001). Similarly, when marmoset monkeys (*Callithrix jacchus*) were presented with two copies of the same object (e.g. A1, A2) they were rewarded for selecting the item on the left, but when two copies of object B (B1, B2) were presented, they were rewarded for selecting the item on the right (Ridley *et al*., 2002). Lesions confined to the anterior thalamic nuclei did not impair performance on this task (Ridley *et al*., 2002). Likewise, rats with anterior thalamic lesions learnt to turn to the right or left depending on which object was placed at the choice point (Sziklas & Petrides, 1999, 2004). Consequently, tasks in this first category seem insensitive to anterior thalamic damage, as found in the present study.

Examples of the second category include evidence that anterior thalamic lesions impair the formation of spatial–visual biconditional associations (Sziklas & Petrides, 1999; Henry *et al*., 2004). In these experiments, rats chose one of two items depending on whether they were located at the north or south of an open-field. Likewise, anterior thalamic lesions impaired an odour-location biconditional task in a circular arena (Gibb *et al*., 2006). Rather like the present task, rats were rewarded for digging in one location when signalled by a medium with a particular odour, but when in a different location the rats were rewarded for digging in a medium with a different odour. Clearly there are strong parallels with the present results (experiments 2D, 2F and 4). There does, however, appear to be an exception to this general pattern when location cues appeared without effect. Rats with anterior thalamic lesions were able to select a particular location in a cross-maze depending on which item was present at the choice point of the maze (Sziklas & Petrides, 2007). Because the task involved approaching the choice point from different directions it was assumed that the rats had successfully used allocentric spatial information to determine the appropriate response (Sziklas & Petrides, 2007). Although this finding might indicate an asymmetry, such that anterior thalamic lesions disrupt biconditional tasks when the signal stimulus is location-specific but not when the conditional response is location-specific (Sziklas & Petrides, 2007), this interpretation is inconsistent with the findings by Gibb *et al*. (2006).

As noted earlier, the present study had two related goals. The first was to compare the profile of anterior thalamic lesion deficits with that following hippocampectomy. Lesions in these two sites seemingly have many of the same effects on tests of spatial and contextual learning (Beracochea *et al*., 1989; Sutherland & Rodriguez, 1989; Aggleton *et al*., 1995; Byatt & Dalrymple-Alford, 1996; Aggleton & Brown, 1999; Warburton *et al*., 2001; Law & Smith, 2012), leading to the notion that they have integrated functions (Aggleton & Brown, 1999). An apparent exception concerns some biconditional tasks as it has been found that hippocampal lesions can impair an egocentric conditional task that is spared by anterior thalamic damage (Sziklas & Petrides, 2004). Likewise, hippocampal, but not anterior thalamic, lesions can impair learning to go to a given location depending on the identity of an object at the choice point (Sziklas & Petrides, 2002, 2007). These findings suggest that anterior thalamic lesions and hippocampal lesions have different profiles of effect when considering spatial biconditional tasks.

The present study strongly supports the opposite view, i.e., lesions in the anterior thalamus and hippocampus have very similar consequences on biconditional discriminations. The present findings could be matched with previous biconditional tasks that used the same test chamber stimuli (hot/cold, checks/spots) as those in the present study. These experiments found that hippocampal lesions, like anterior thalamic lesions, spare acquisition of the biconditional rule (Coutureau *et al*., 2002). Likewise, prior experiments using the same biconditional digging task as that in the present study found that hippocampal lesions spared the ability to use local contextual cues to learn in which cup to dig for food rewards (Albasser *et al*., 2013). Then, just as in the present study, hippocampectomy impaired the ability to use room location cues to determine in which cup to dig (Albasser *et al*., 2013). The similarity between the impact of anterior thalamic and hippocampal lesions extended to the ability to learn a go/no-go place discrimination when trained by running in one direction (Albasser *et al*., 2013; experiment 3B present study), but then performing poorly on a subsequent biconditional that used this same spatial information. Consequently, the profiles of spared and impaired performance following anterior thalamic and hippocampal lesions on the tasks used in the present study appear extremely similar. Furthermore, others have also demonstrated that rats with hippocampal lesions are unable to form object-location and odour-location associations using distal room cues (Gilber & Kesner, 2002); however, these same rats were able to solve a non-spatial biconditional problem that involved object–odour associations. These results are also consistent with those reported in the present communication following anterior thalamic damage, once again highlighting the similarity between hippocampal and anterior thalamic function for biconditional learning. This conclusion closely accords with two disconnection studies showing that the hippocampus and anterior thalamic nuclei function together to solve the visuospatial biconditional problem, select object A at the north of an arena but select object B at the south end (Henry *et al*., 2004; Dumont *et al*., 2010).

The matching patterns of learning found after hippocampal (Albasser *et al*., 2013) and anterior thalamic lesions for the biconditional discriminations that involved either local contextual cues (spared) or distal location information (impaired) highlight the need to understand what qualitatively separates contextual cues from spatial cues. One potential difference concerns their proximity. Local cues, including visual cues, can be regarded as being available by direct exploration. That is, the cues are within the rat’s ‘working space’ and, hence, not further than the tip of the nose or the vibrissae (Parron *et al*., 2004). There are, however, shortcomings with this proximal–distal distinction when trying to explain the present set of results. One problem is that the rats with anterior thalamic lesions could still acquire a spatial go/no-go task that relied on distal cues (experiment 3B, Fig. 8D). Furthermore, rats with anterior thalamic lesions can readily solve visual discriminations in a water maze in which the stimuli were selected from a distance (Aggleton *et al*., 2009; see also Ridley *et al*., 2002). There also remains the problem of deciding *a priori* when a cue is ‘distal’ and when it is ‘proximal’ (Good *et al*., 1998).

A different explanation focuses on the nature of the stimuli used in the various experiments. For the biconditional problems in the automated test chamber (experiment 1) and the test boxes with different appearances (experiment 2E), which the ATNx1 rats could readily solve, the critical stimuli could be discriminated by their individual salient features (e.g. different wall patterns, floor temperatures and floor coverings). As a consequence, any stimulus ambiguity from overlapping or common elements was kept low. In contrast, in those tasks where anterior thalamic lesions impaired performance (experiments 2D, 2F, 3A, 3B and 4), the rats had to use distal room cues to identify locations where presumably there would be overlap of common cues. This potential requirement to disambiguate common cues closely relates to the notion that the hippocampus is required for contextual learning when it inherently involves configural learning, reflecting the need to distinguish overlapping cues and utilise pattern separation (Gaffan & Harrison, 1989; Gilbert *et al*., 1998; Holland & Bouton, 1999; Rudy, 2009; Iordanov *et al*., 2011). Such functions might be expected to depend on the integrity of the extended hippocampal system, including the anterior thalamic nuclei, and so help to explain the present pattern of results. One problem with this account, however, concerns the variable effects of hippocampal lesions on configural tasks. Given the previous account, it might be supposed that visual configural tasks are consistently hippocampal-dependent, yet this is sometimes not the case (Rudy & Sutherland, 1995; Sanderson *et al*., 2006; Saksida *et al*., 2007).

One possible solution is to suppose that the hippocampal–anterior thalamic axis is important for a subset of configural problems. These problems require configural learning, but also involve determining the relative spatial positions of the common cues. Such configural problems require ‘structural learning’ as the animal has to learn not only which elements are combined in a given scene but also how these elements are positioned with respect to each other, i.e., how they are structured (George *et al*., 2001; George & Pearce, 2003). There is appreciable evidence that the hippocampus is required for this form of learning (Save *et al*., 1992; Aggleton & Pearce, 2001; Sanderson *et al*., 2006; Barker & Warburton, 2011; Albasser *et al*., 2013). While there is also evidence that the anterior thalamic nuclei are required for tests that should tax structural learning (Parker & Gaffan, 1997; Wilton *et al*., 2001), anterior thalamic lesions failed to impair a formal test of this form of learning (Aggleton *et al*., 2009).

An alternative, closely related, proposal concerns the role of the hippocampus for pattern separation (Gilbert *et al*., 1998; Hunsaker & Kesner, 2013; Leutgeb *et al*., 2007; Rolls & Treves, 1994). This process would help the animals to distinguish room cues when they overlap, a situation presumably more prevalent in the bidirectional than unidirectional tasks. Consequently, this account would explain the different results for the two types of go/no-go tasks. This account would also have to assume that pattern separation depends on anterior thalamic interactions with the hippocampus, an assumption that is not implausible given the results of disconnection studies (Dumont *et al*., 2010; Henry *et al*., 2004; Warburton *et al*., 2001). It is not clear, however, how a pattern-separation account will explain the failure to learn the biconditional problem (experiment 4) once the relevant location cues had been distinguished. While it could be argued that this additional biconditional deficit reflects the conflict between competing similar demands on the rats in the biconditional task, exaggerated by the use of spatial stimuli, this account remains largely *post hoc* in nature. A further possibility is that the deficit reflects the combination of pattern separation demands and a closely related mnemonic component (Hunsaker & Kesner, 2013).

This consideration of spatial learning brings us to the second goal of the present study, namely, to identify the nature of any observed biconditional learning deficits associated with anterior thalamic damage. As already noted, the use of distal spatial cues appears to be a common factor in many examples of biconditional tasks sensitive to anterior thalamic damage (Gibb *et al*., 2006; Henry *et al*., 2004; Sziklas & Petrides, 1999). One apparent exception, seemingly unaffected by anterior thalamic lesions, involved using object identity to signal whether to go to place A or place B for reward (Sziklas & Petrides, 2007). The goal of the final test (experiment 4) was, therefore, to understand more precisely what prevents rats with anterior thalamic lesions from solving most biconditional discriminations involving distal spatial cues. Consequently, rats were first trained on a spatial go/no-go task (experiment 3B). When measured by latency scores, the anterior thalamic lesions disrupted performance, resulting in more rapid response times overall. There is, however, a concern that this thalamic surgery can induce hyperactivity (Jenkins *et al*., 2004; Poirier & Aggleton, 2009) and so latency ratios might be more appropriate. It was, therefore, notable that the ATNx2 group seemed unimpaired when location discrimination performance was measured as a ratio of go/no-go latencies (Fig. 8D). However, despite learning this go/no-go location task, the thalamic-lesioned rats were still unable to use that same spatial information to guide a subsequent biconditional learning task (experiment 4). In fact, the rats with anterior thalamic lesions remained at chance (see Fig. 9). This dissociation clearly questions the parsimonious notion that it is the discrimination of distal location information *per se* that accounts for the pattern of anterior thalamic lesion deficits in the present study. Rather, the added burden of the biconditional problem left the ATNx2 rats at chance.

The present results strengthen the notion that the anterior thalamic nuclei and hippocampus work together to resolve spatial problems, including biconditional discriminations (see also Warburton *et al*., 2000, 2001; Henry *et al*., 2004). Given the dense, direct fornical projections from the hippocampus to the anterior thalamic nuclei it might naturally be supposed that fornix lesions would, therefore, match the impact of anterior thalamic damage on biconditional learning tasks. There are, however, problems with this prediction. Not only has it been found that anterior thalamic and fornix lesions can both spare spatial biconditional problems (Sziklas & Petrides, 2002, 2007) that are sensitive to hippocampectomy (Sziklas & Petrides, 2002), but of more concern is the finding that fornix lesions spare a spatial biconditional task (Dumont *et al*., 2007; Sziklas *et al*., 1998) that is sensitive both to anterior thalamic lesions (Sziklas & Petrides, 1999) and to crossed anterior thalamic–hippocampal lesions (Henry *et al*., 2004). Such findings (see also Aggleton *et al*., 2009; Warburton & Aggleton, 1999) either suggest the importance of indirect routes linking the hippocampus with the anterior thalamus, e.g. via the retrosplenial cortex (Vann *et al*., 2009), or indicate that critical thalamic contributions involved in biconditional learning emanate from the diencephalon and then target the hippocampus and, hence, do not require the fornix (Taube, 2007; Vann, 2009; Vann & Albasser, 2009). Both interpretations could be correct. In order to test the former notion, combined lesions were placed in the retrosplenial cortex and fornix, resulting in impaired learning of a spatial biconditional task otherwise spared by fornix lesions alone and by retrosplenial lesions alone (Dumont *et al*., 2007, 2010; St-Laurent *et al*., 2009; Sziklas *et al*., 1998). Such findings highlight the need to uncover the various pathways by which the anterior thalamic nuclei and the hippocampus might conjointly support forms of biconditional learning.

## Abbreviations

ATN: anterior thalamic nuclei
